# Constrictive pericarditis complicating cardiac transplantation

**DOI:** 10.1186/s13019-015-0314-x

**Published:** 2015-08-25

**Authors:** Affan Umer, Nauman Khalid, Lovely Chhabra, Sarfaraz Memon, David H. Spodick

**Affiliations:** 1 Department of Surgery, Saint Francis Hospital and Medical Center, University of Connecticut Health Center, Hartford, CT USA; 2 Hartford Hospital, University of Connecticut Health Center, Hartford, CT USA; 3 Saint Vincent Hospital, University of Massachusetts Medical School, Worcester, MA USA

**Keywords:** Constrictive pericarditis, Pericardiectomy, Cardiac transplant

## Abstract

Constrictive pericarditis is a disease characterized by progressive pericardial fibrosis. If left untreated it can lead to progressive heart failure and can be severely disabling. Medical management with non-steroidal anti-inflammatory drugs in combination with colchicine is promising in the acute phase of the disease but for more chronic cases pericardiectomy offers the best chance for hemodynamic recovery. Constrictive pericarditis after cardiac transplantation is a rare phenomenon. Current literature suggests that early pericardiectomy may be the most effective treatment in this subset of patients as well.

## To the Editor

We read with great interest the recently published article byZhu et al. in the Journal of Cardiothoracic Surgery [1]. The authors have provided valuable insight regarding the role of pericardiectomy in constrictive pericarditis. Their mortality rate of 5.4 % falls on the lower end of the spectrum in reported literature and their one year survival rate of 92 % speaks volume of the excellent results that can be achieved in high volume centers [1]. However, we wish to highlight a few important points relevant to the article.

A relatively newer and less known development of constrictive pericarditis is seen recently after cardiac transplantation. This is an unusual presentation as the transplanted heart is believed to be free of any pericardial tissue. The data on this entity is very limited but judging from the few published case reports, pericardiectomy offers the only chance of successful physiologic recovery in this subset of patients [2, 3]. We would like to inquire whether Zhu and colleagues encountered patients with post cardiac transplant constrictive pericarditis in their cohort in the subgroups including indeterminate (*n* = 106 patients) and post-surgery (*n* = 19 patients) as indicated in Table one of their manuscript [1].

Pericardiectomy is a well established treatment for sub acute to long-standing constrictive pericarditis [4]. However, a subset of patients affected with acute constrictive pericarditis may only experience transient constriction and may benefit from only medical management with non-steroidal anti-inflammatory drugs and/or colchicine for 2 to 3 months duration. We wonder if a similar therapeutic algorithm was used in their study especially in the patients presenting with acute constrictive physiology? We would appreciate if the authors could share their invaluable experience on these subgroup of patients.
